# The Effect of Evening Technology Use on Objective Sleep in Older Adults: Protocol for a Crossover Randomized Controlled Trial

**DOI:** 10.2196/84512

**Published:** 2026-01-30

**Authors:** Sarah Nauman Ghazi, Anders Behrens, Joakim Niklasson, Johan Sanmartin Berglund, Peter Anderberg

**Affiliations:** 1 Department of Health, Blekinge Institute of Technology, Karlskrona, Sweden, 46 455385499

**Keywords:** sleep, technology use, older adults, cross-over randomized controlled trial, gerontechnology, eHealth.

## Abstract

**Background:**

Evening technology use (ETU) has been associated with sleep disturbances, often attributed to blue light exposure and cognitive arousal. However, most of the existing evidence focuses on younger populations and relies primarily on subjective measures. As older adults increasingly engage with both passive and active technology use, it is important to investigate how ETU impacts objective sleep. Currently, there is also a limited understanding of how particular evening digital activities, especially active versus passive engagement, affect objective sleep in older adults.

**Objective:**

This study aims to investigate the impact of exposure to ETU on both objective and subjective sleep outcomes in older adults.

**Methods:**

This is a randomized crossover trial involving approximately 55 adults aged 60‐75 years from the ongoing Swedish National Study on Aging and Care – Blekinge. Each participant will undergo 3 one-week intervention periods: active ETU, passive ETU, and a nondigital activity (book reading), with one-week washout periods in between. The order of interventions will be randomized. Sleep will be assessed using a home-based electroencephalography device (MUSE headband) and daily self-reports. Primary outcomes are sleep onset latency and wake after sleep onset. Secondary outcomes include objective measures such as total sleep time, sleep efficiency, and time spent in REM, deep, and light sleep, subjective sleep quality, adherence, and perception of the intervention and comfort of using the objective measurement tool, that is, the electroencephalography headband. Linear mixed-effects models (with fixed effects for condition and period and a random participant intercept) were used to analyze crossover effects on sleep outcomes.

**Results:**

Participant recruitment and data collection began in the fall of 2025 and will continue through summer 2026 or until the target sample size is reached. Data collection is scheduled to be completed by spring 2027. Results will include participant flow, baseline characteristics, adherence data, and comparative analyses of the 3 intervention conditions. Within-subject statistical models will be used to evaluate differences in sleep outcomes and investigate the associations between ETU and sleep quality.

**Conclusions:**

This crossover study will clarify how active and passive ETU, compared with a nondigital activity, relate to objective sleep in older adults. Findings will inform simple, practical recommendations for technology use before bed in late life.

## Introduction

The digital society has made it easy for its citizens to use technology for multiple purposes, from everyday tasks such as banking to entertainment, relaxation, and promoting health. Recent data indicate that about 90% of Swedes aged 60 years and older use the internet, with approximately 75% using the internet daily, showcasing digital engagement in later life [1], raising important questions about how this use may influence restorative behaviors such as sleep. In particular, evening technology use (ETU) before bed often becomes a routine; therefore, it is important to clarify its effects on sleep in order to protect sleep health among older adults.

Sleep is an important determinant of health and well-being, particularly in older adults, who may experience changes in sleep architecture and quality due to aging and lifestyle factors [2]. Even factors such as technology use may shape these sleep patterns. The increasing use of technology within the general population and among older adults [1], especially ETU and its potential effects on sleep, has raised concerns among health care professionals and researchers. Traditionally, these concerns have focused on the impact of bright light or blue light on melatonin release, which can disrupt the circadian rhythm. However, this unidirectional view has been refined by a bidirectional model that recognizes that technology can disrupt sleep and vice versa through multiple mechanisms. These include sleep time displacement, the arousal hypothesis, and the bright light hypothesis. Consequently, poor sleep can also lead to increased technology use, as it fills the time and provides cognitive distraction [3]. According to the arousal hypothesis proposed by Bauducco et al [3], using engaging or active digital content close to bedtime (eg, video games or social media exchanges) increases a person’s physiological and psychological arousal by increasing heart rate, alertness, or stress levels before bed. This, in turn, makes it harder to fall asleep quickly or stay asleep.

Existing research also highlights the influence of digital device use on sleep through mechanisms such as blue light exposure [4], cognitive stimulation [5], and emotional arousal [6,7]. Much of the current research on technology and sleep focuses on children, adolescents, and younger adults [8-10]. Far fewer studies have investigated whether and how these mechanisms generalize to older adults.

Older adults use technology for social interaction [11], entertainment [12], cognitive exercises [13], and overall well-being [14]. Older adults between the ages of 60 and 75 years are often overlooked in current research on sleep. This age group is old enough to experience age-related changes in sleep patterns, yet young enough to minimize the effects of frailty, multiple health conditions, and the use of multiple medications, which could skew the results. Additionally, their experiences with technology and sleep may differ from those of younger populations [15].

Additionally, little is known about how the type of ETU, whether passive (such as watching a documentary) or active (such as playing a video game), affects sleep compared with traditional, nondigital presleep habits. There has been some research that suggests that watching nature videos can promote relaxation, reduce stress, and have positive effects on cognitive and emotional health [10], which are factors that could positively influence sleep quality. Furthermore, it has also been shown that engaging in complex cognitive tasks before sleep can significantly improve both objective and subjective sleep quality, particularly for individuals who typically struggle with sleep issues [11]. On the other hand, video gaming before bed is associated with delayed sleep onset in adolescents [7]. Conversely, some research suggests that casual gaming may not significantly disrupt sleep, indicating that the type and intensity of gaming matter [8,16]. Moreover, to measure sleep objectively is important as it provides reliable, instrument-based evidence of sleep health that complements subjective reports and reduces bias [17,18]. Therefore, the aim of the study is to investigate the impact of exposure to ETU on both objective and subjective sleep outcomes in older adults.

## Methods

### Objectives

### Study Design

This study adopts a crossover randomized controlled trial (RCT) to examine how ETU influences objective and subjective sleep in older adults aged 60 to 75 years. Three conditions will be compared: (1) passive ETU, (2) active ETU, and (3) nondigital activity.

After completing an initial baseline assessment via survey, participants will undertake a 5-week schedule composed of 3 separate, one-week intervention periods. At baseline, each participant is randomized to one of six possible sequences of the 3 conditions (passive ETU, active ETU, nondigital; 3!=6). Participants then complete 3 one-week intervention periods in their assigned order, separated by 2 one-week washouts (total duration: 5 wk). We chose a 7-day washout period because systematic reviews of digital detox interventions often recommend this duration for detoxification [19].

During each intervention week, participants will maintain a daily evening log to record subjective sleep quality, ETU, and the comfort of using a wearable headband. Objective sleep outcomes will be assessed using electroencephalography (EEG) collected via a headband (Muse-S Athena, InteraXon, Toronto, ON, Canada), while self-reported logs capture subjective experiences. This crossover design allows for within-subject comparisons, thereby reducing variability, controlling for individual differences in sleep patterns, and confounding factors, while enhancing statistical power.

### Population and Recruitment

Participants in this study will be recruited from the Swedish National Study on Aging and Care – Blekinge (SNAC-B) cohort. SNAC-B is part of a large, national, population-based research initiative in Sweden focused on individuals aged 60 years and older [20]. The study is conducted at the Research and Development Clinic affiliated with the Department of Health at Blekinge Institute of Technology (BTH).

The SNAC-B population offers a well-characterized cohort of older adults, increasing the likelihood of identifying individuals who meet the inclusion criteria for this study. Eligible participants will be directly contacted by the research team, ensuring both transparency and voluntary participation. Detailed study information will be provided before obtaining informed consent.

Participants eligible for inclusion in the study will be aged between 60 and 75 years and must be regular users of digital devices such as smartphones or tablets. They should be willing and able to use an EEG headband for sleep monitoring throughout the intervention period. Additionally, participants must be able to take part in either the RCT, follow-up interviews, or both, depending on study needs and personal preference.

Individuals will be excluded from participation if they have a diagnosed sleep disorder or severe cognitive impairment. Use of medications that significantly influence sleep, such as melatonin or benzodiazepines, will also serve as an exclusion criterion.

Eligible participants aged 60‐75 years will be identified from the SNAC-B cohort. A random number will be generated using Excel to generate a random order of potential participants, who will be contacted sequentially until the target sample size of 55 is reached. One investigator (SNG) generated the randomization list for the 6 possible condition sequences using computer-generated random numbers in Excel. Random numbers, not participant IDs, determined assignment. Sequence codes were kept in a secure file on the university’s server and accessed only by the authors of this study.

Before analysis, a coauthor not involved in analysis (AB) will replace all participant IDs with pseudonymous IDs; the ID key is stored separately. AB will also remap the 3 conditions to neutral labels (eg, X/Y/Z). The primary analysts (SNG and JN) will conduct the primary analyses on masked labels and pseudonymous IDs. Unmasking will occur only after the analysis code is finalized and the results are locked.

The sample size is determined through performing a priori power analysis, conducted using effect size estimates from Leger et al [21] on evening screen use and sleep in older adults. Details of power analysis are shown in Multimedia Appendix 1. Recruitment will proceed in waves of 10 participants for logistical feasibility. All enrolled participants will complete all 3 intervention conditions in a randomized order.

### Intervention

#### Overview

**Figure 1.**
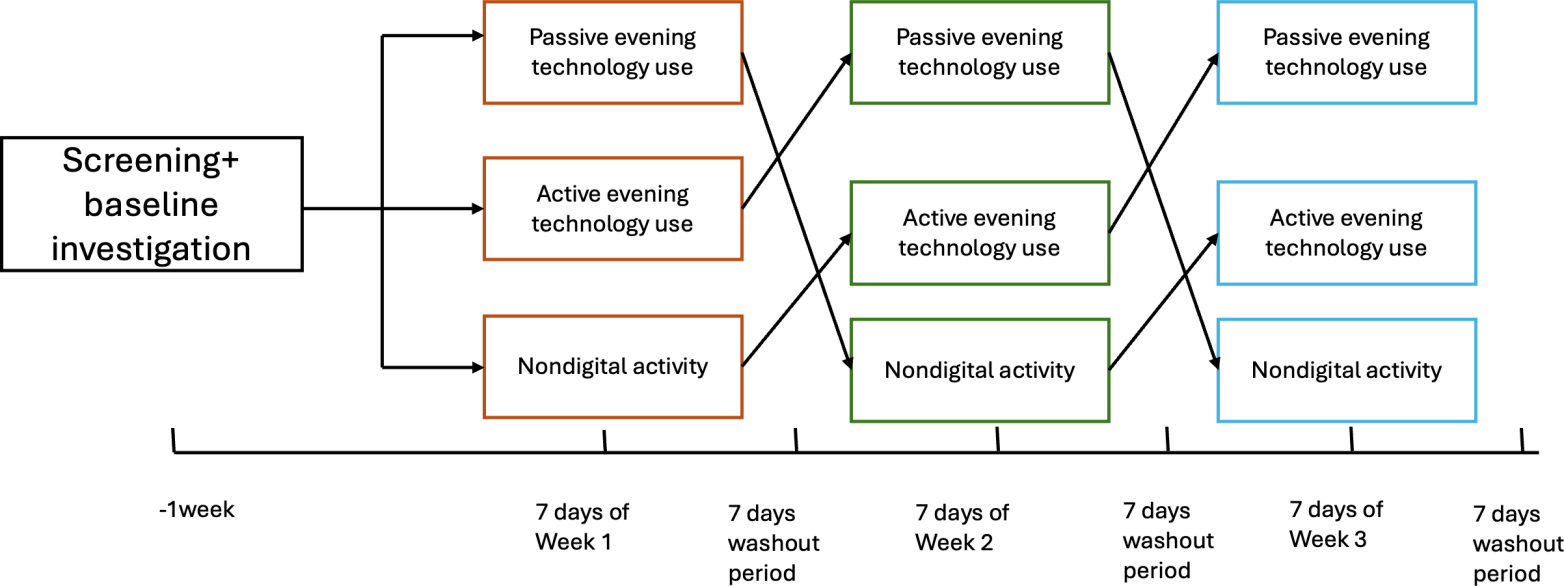
Schematic of the randomized crossover trial design. Participants complete three 1-week conditions in randomized order, separated by 1-week washout periods, following baseline investigation.

This study uses a crossover RCT design in which participants progress through 3 different intervention conditions: passive ETU, active, and nondigital activity, in a randomized order. Each intervention phase lasts for one week, followed by a one-week washout period to reduce carryover effects, resulting in a total study duration of 5 weeks (Figure 1).

#### Baseline Assessment

Before the first intervention phase, participants complete a baseline survey (in Swedish language) assessing sociodemographic characteristics, subjective sleep health, chronotype, lifestyle factors, sleeping habits, general health status, and technology use (Multimedia Appendix 2).

#### Main Intervention

Participants will engage with the assigned evening activity for 30‐60 minutes within the final 60 minutes before intended bedtime. To minimize variability in the interventions, the digital activities are standardized. To reduce the likelihood of accidentally falling asleep, participants should complete the evening activity seated (not lying in bed) and move to bed only when ready for lights-out. If they fall asleep during the activity before starting the MUSE recording, they should start the recording as soon as they notice and select “fell asleep before recording” in the morning survey. For all technology sessions, participants will set the device display to medium brightness (≈50% of the slider) at the start of the activity and maintain this setting throughout. Auto/adaptive brightness should be turned off to prevent fluctuations. The activity may continue until the participant is ready to sleep (lights-out). Immediately before lying down to sleep, participants will put on the MUSE headband, start the recording, then switch off the lights and attempt to sleep. No additional screen use is permitted after recording starts. Compliance will be checked through self-reporting in the daily log.

#### Passive Evening Technology Use

Participants watch a curated selection of soothing documentaries, those narrated by Sir David Attenborough or the secrets of the forest on SVT Play, for 30‐60 minutes each evening, one hour before bedtime, using smartphones or tablets.

#### Active Evening Technology Use

Participants engage with the interactive word-based game Ruzzle for 30‐60 minutes each evening, one hour before bedtime [2], using smartphones or tablets. It is to provide mental stimulation and engage participants actively.

#### Nondigital Activity

Participants read a nonfiction book of their choice for the same duration, avoiding all digital screens for one hour before bedtime.

#### Daily Logs

Throughout each intervention week, participants maintain a daily log (in Swedish) of their intervention adherence, morning assessments of subjective sleep quality, and the feasibility or comfort of using the EEG headband. The daily survey also includes an open-ended item asking whether anything unusual since the prior evening could have affected sleep, with examples written such as more caffeine than usual (especially after ≈4 PM) and alcohol (Multimedia Appendix 2).

### Measures

The baseline and daily log surveys, along with the sociodemographic and subjective measures, are attached in MultimediaAppendices 2  3.

#### Sociodemographic and General Measures

The included variables were age, gender, living status, economic situation, employment situation, education, health status, sleep health scores, and the reduced Morningness–Eveningness Questionnaire (for chronotype) [22,23] (MultimediaAppendices 2  3 contain detailed questions).

#### Primary Outcome Measures

##### Overview

The primary outcomes of this study focus on changes in objective sleep onset latency (SOL) and wake after sleep onset (WASO) assessed using objective data collected via the MUSE EEG headband. This wearable device records brainwave activity throughout the night, enabling detailed analysis of various sleep parameters. The primary sleep parameters are provided below.

##### Sleep Onset Latency (SOL)

This refers to the duration of time it takes for a participant to transition from full wakefulness to sleep. Sleep latency will be automatically calculated by the MUSE EEG headband as the time from the start of recording to the first detected epoch of NREM sleep.

##### Wake After Sleep Onset (WASO)

WASO measures the cumulative amount of time (in minutes) a participant spends awake after initially falling asleep until final morning awakening. Elevated WASO values may indicate fragmented or disrupted sleep, which can negatively affect sleep quality and daytime functioning. It is automatically computed by summing detected wake periods within the sleep period time by the MUSE device algorithm.

### Secondary Outcomes Measures

#### Overview

In addition to the objective measures of sleep, the study will assess several secondary outcomes that capture participants’ subjective experiences, intervention adherence, and device usability.

#### Total Sleep Time (TST)

Total sleep time (TST) is the total amount of time spent asleep during the night, excluding any periods of wakefulness after sleep onset. It reflects the overall quantity of sleep and is a core indicator of sleep sufficiency. Derived directly from the MUSE’s sleep stage classification, summing up the total minutes spent in light, deep, and REM sleep stages.

#### Sleep Efficiency (SE)

Sleep efficiency (SE) is the percentage of time in bed actually spent sleeping. It is calculated by dividing TST by the total time spent in bed and multiplying by 100. Higher SE suggests less wakefulness during the night and better overall sleep quality. SE is calculated automatically by the MUSE device algorithm.

#### Sleep Stages

The EEG data will be analyzed to determine the time spent in different sleep stages: REM (rapid eye movement) sleep, deep sleep (slow-wave sleep), and light sleep. These are expressed in percentages.

#### Subjective Sleep Quality

Each morning, participants will complete a low-burden Single-Item Sleep Quality Scale, a validated tool designed to assess overall perceived sleep quality [24]. We chose this brief item to minimize burden and support daily completion in older adults. This brief measure captures participants’ evaluations of how well they slept the previous night, ranging from 0 (terrible) to 10 (excellent). Subjective ratings are analyzed alongside objective EEG outcomes as complementary indices of sleep and are tested across passive ETU, active ETU, and nondigital activity.

#### Intervention Adherence and Perception

Participants will be asked whether they were able to complete the assigned evening activity the previous night. If the answer is “yes,” they will also report how long they engaged in the intervention (in minutes). This self-report measure will help evaluate the feasibility and real-world compliance with each intervention condition.

To assess how participants perceived each evening activity, they will complete a brief daily rating scale immediately after the intervention. The scale includes 5 items rated from 0 (not at all) to 10 (extremely), adapted from established tools: the Visual Analog Mood Scales for calmness [25], the Positive and Negative Affect Schedule for enjoyment and stress [26], and the User Engagement Scale for engagement and perceived meaningfulness [27]. This approach allows for low-burden, repeated assessment of affective and cognitive responses relevant to sleep-related outcomes in older adults. The survey is included in MultimediaAppendices 2  3. The internal consistency of the items (Cronbach α), once the data is collected, will be reported in the main study.

#### Comfort Level of EEG Headband

To assess usability and participant burden, participants will rate the comfort of wearing the EEG headband during the night using a 5-point Likert scale. The scale ranges from 1 (very uncomfortable) to 5 (very comfortable). This data will inform the acceptability of the MUSE EEG headband as a sleep monitoring tool and identify any potential discomfort that may influence sleep or compliance.

### Statistical Analysis Plan

Analyses will be conducted in SPSS Statistics (version 29.0.x; IBM Corp, Armonk, NY); the exact patch (eg, 29.0.2) will be reported at analysis. Descriptive statistics will be used to summarize demographic information, baseline sleep characteristics, chronotype prevalence, overall intervention adherence (percentage of nights completed; minutes engaged), perception ratings (calmness, stress, engagement, enjoyment, meaningfulness on 0‐10), and EEG headband comfort (1‐5 Likert), presented as mean (SD) or median (IQR), as appropriate.

Primary and secondary sleep outcomes will be derived from MUSE EEG auto-scoring using the device’s standard algorithm. This study does not include manual rescoring or a new polysomnography comparison; instead, we rely on published validation studies demonstrating agreement with polysomnography/manual scoring [28,29]. All analyses will use participant-level week means (nightly values averaged within each intervention week/condition). Before analysis, we will check data quality. A night is classified as VALID if the device records ≥4 hours within the main sleep window and yields plausible values (eg, TST 2‐12 h, SOL 0‐180 min, WASO 0‐240 min); otherwise, it is INVALID. All the valid nights will be included in the analysis. During the analysis, the data analyst will be blinded to intervention allocation. Unblinding will occur only after the analysis code is finalized, outputs are generated, and the primary results are locked. Participant-level week means (nightly values averaged within each intervention week/condition) will be calculated for descriptive purposes.

The coprimary outcomes are SOL and WASO derived from the MUSE EEG. The primary objective compares combined ETU versus nondigital activity, where ETU is defined by collapsing the 2 technology conditions (passive and active) into a single ETU category. This will be tested as a prespecified linear contrast of the condition marginal means from a linear mixed-effects model (LMM). For each primary outcome (SOL and WASO), we will fit an LMM with fixed effects for condition and period and a participant-specific random intercept. The LMM analyses will use all available valid nights. In addition to the prespecified combined-ETU contrast, we will estimate all pairwise condition contrasts (active vs passive, active vs nondigital, passive vs nondigital) using the Tukey adjustment. Model-based estimates, 95% CIs, and *P* values will be reported, together with a standardized within-participant effect size. Multiplicity control across the 2 coprimary endpoints (SOL and WASO) will be performed using the Holm procedure with a family-wise α of .05 (2-sided). If at least one coprimary endpoint is statistically significant after the Holm adjustment, we will proceed to confirmatory testing of the prespecified key secondary outcomes; otherwise, these secondary outcomes will be reported as exploratory. Model assumptions will be checked; if needed, outcomes will be transformed or analyzed using an appropriate generalized mixed model.

Key secondary objective outcomes are TST, SE, and sleep stages (REM%, Deep% [SWS], Light%). These outcomes will be analyzed using the same LMM framework (fixed effects for condition and period; random participant intercept), with Tukey adjustment for pairwise condition comparisons. To control for multiplicity across the key secondary outcomes, *P* values will be adjusted using Holm’s procedure (2-sided α=.05), applied only if the gatekeeping criterion on the coprimary outcomes is met. All remaining outcomes (eg, subjective sleep, adherence, and comfort) will be treated as exploratory and interpreted primarily via effect estimates and 95% CIs.

We will briefly explore how subjective sleep aligns with objective measures using simple descriptive statistics (mean, SD and median, IQR). The full analysis code will be included as a supplementary file (including SPSS syntax and output) when the main study is reported. The statistical plan for each research question is presented in Table 1.

**Table 1.** Research questions with hypotheses, planned analysis, and expected endpoints.

Objectives	Research question	Hypothesis (H₁)	Null hypothesis (H₀)	Endpoints and analysis
Primary objective	Does evening technology use (ETU) affect primary objective sleep outcomes (WASO^a^ and SOL^b^) compared with engaging in nondigital activity like reading physical books?	Older adults exposed to ETU before bedtime will show significant impairments in primary objective sleep outcomes than those exposed to nondigital activity.	Older adults exposed to ETU before bedtime will show no significant impairments in primary objective sleep outcomes than those exposed to nondigital activity.	Primary outcomes: SOL, WASO (MUSE EEG)^c^. Linear mixed-effects model with fixed effects for condition and period and a random participant intercept. Multiplicity across SOL and WASO controlled using Holm (α=.05, 2-sided). Pairwise condition comparisons reported with Tukey adjustment. Report estimated marginal mean 95% CIs, *P* values, and Cohen *d* (standardized within-participant effect sizes).
Key secondary	Does passive ETU^d^ lead to better objective sleep outcomes than active ETU?	Passive ETU will show significant differences in sleep outcomes than active ETU.	Passive ETU will lead to no difference in sleep outcomes than active ETU.	SOL, WASO, SE^e^, TST^f^, Sleep Stages (REM%^g^, Deep%, Light%). LMM^h^ with fixed effects for condition and period and a random participant intercept. Confirmatory testing follows a hierarchical (gatekeeping) strategy: these outcomes are formally tested only if ≥1 coprimary endpoint is significant after Holm adjustment. Multiplicity across key secondary outcomes controlled using Holm (α=.05). Pairwise condition comparisons use Tukey adjustment.
Key secondary	Does passive ETU lead to better objective sleep outcomes than nondigital activity^i^?	Passive ETU will show significant differences in sleep outcomes than nondigital activity.	Passive ETU will show no significant differences in sleep outcomes than nondigital activity.	SOL, WASO, SE, TST, Sleep Stages (REM%, Deep%, Light%). LMM with fixed effects for condition and period and a random participant intercept. Confirmatory testing follows a hierarchical (gatekeeping) strategy: these outcomes are formally tested only if ≥1 coprimary endpoint is significant after Holm adjustment. Multiplicity across key secondary outcomes controlled using Holm (α=.05). Pairwise condition comparisons use Tukey adjustment.
Key secondary	Does active ETU^j^ lead to better objective sleep outcomes than nondigital activity?	Active ETU will show significant differences in sleep outcomes than nondigital activity.	Active ETU will show no significant differences in sleep outcomes than nondigital activity.	SOL, WASO, SE, TST, Sleep Stages (REM%, Deep%, Light%). LMM with fixed effects for condition and period and a random participant intercept. Confirmatory testing follows a hierarchical (gatekeeping) strategy: these outcomes are formally tested only if ≥1 coprimary endpoint is significant after Holm adjustment. Multiplicity across key secondary outcomes controlled using Holm (α=.05). Pairwise condition comparisons use Tukey adjustment.
Key secondary	Are there differences in subjective sleep quality across passive ETU, active ETU, and nondigital activity?	Subjective sleep quality will differ across conditions.	Subjective sleep quality does not differ across conditions.	Single-Item Sleep Quality (0‐10). Exploratory Analysis using LMM with fixed effects for condition and period (and sequence if included) and a random intercept for participant. Report model-based estimated marginal means for each condition and pairwise contrasts with Tukey-adjusted 95% CIs (*P* values reported descriptively)
Secondary	What do participants’ adherence to the assigned evening activity and their perceived experience of the activity look like over the study?	Dropout and nonadherence rates will be higher in the active ETU condition compared with passive and nondigital activity.	Dropout and nonadherence rates are equal across all 3 conditions.	Adherence: % of nights with ≥30 min activity; mean engagement time. Exploratory Analysis using LMM with fixed effects for condition and period (and sequence if included) and a random intercept for participant.
Implementation	What is the overall comfort/usability of the MUSE EEG headband during sleep?	(Exploratory, no formal hypothesis)	Not applicable	Comfort rating (1‐5 Likert). Exploratory/descriptive only. Report mean (SD), median (IQR), % nights rated ≥4.

a WASO: wake after sleep onset.

b SOL: sleep onset latency.

c EEG: electroencephalography.

d Passive ETU: passive evening technology use (watching a documentary on SVT PLAY app).

e SE: sleep efficiency.

f TST: total sleep time.

g REM: rapid eye movement.

h LMM: linear mixed-effects model.

i Nondigital activity (reading a book).

j Active ETU: active evening technology use (playing a video game called Ruzzle).

### Ethical Considerations

The study will adhere to ethical standards as outlined by the Swedish research ethics framework. Ethical approval was obtained from the Regional Ethical Review Board prior to study initiation (Dnr 2025-02006-01). All participants will receive comprehensive information about the study’s aims, procedures, potential risks, and their rights before providing written informed consent.

Participant confidentiality will be maintained through strict data management procedures. All data will be stored securely, ensuring that individual identities cannot be traced. All study data are stored on an encrypted, firewall-protected BTH server. Records are pseudonymized; identifiers are not stored with research data. Participation in the study is entirely voluntary, and participants may withdraw at any time without consequence. Procedures comply with GDPR, including participants’ rights to access, rectification, and deletion.

## Results

Participant recruitment and data collection commenced in the Fall of 2025. Recruitment is ongoing and will continue through Summer 2026 or until the target sample size is reached. The completion of data collection is projected for Spring 2027. We hypothesize that older adults exposed to ETU before bedtime will show significant differences in primary objective sleep outcomes compared with those exposed to nondigital activity. We also hypothesize that Passive and Active technology use will show significant differences in sleep outcomes among themselves and versus nondigital activity.

In the final trial report, the following data will be presented.

1. Number of participants screened, enrolled, randomized, and completed each intervention phase
2. Reasons for dropouts, exclusions, and nonadherence during any of the 3 conditions
3. Baseline characteristics of all the variables measured
4. Results of the statistical analysis based on the research questions of the primary and secondary outcome measures

## Discussion

### Principal Findings

This study aims to evaluate how ETU and types of evening activities, specifically Active technology use, Passive technology use, and nondigital activity, affect both objective sleep parameters and subjective sleep quality in older adults. In this 3-period crossover study of older adults, we hypothesize that, compared with a nondigital presleep activity, both passive and active ETU will be associated with longer SOL and greater WASO on EEG. We further expect active technology use to show the largest effects. As a secondary expectation, subjective sleep quality ratings will be lower on technology-use nights and will broadly follow the objective patterns.

A previous study revealed in OA that screen use before bed is positively associated with subjective sleep health [15], contrary to the conventional opinion that screen use has a negative relationship in the younger population [30-32]. This variability is an empirical motivation to test whether effects differ by activity type. Our within-person crossover study compares active and passive ETU to a nondigital activity, allowing us to determine if any effects are specific to the activity without inferring mechanisms. Evening activities can affect presleep arousal through 2 main routes: light exposure from screens (blue-enriched light) [3,33] and cognitive/emotional engagement [5]. The study expects that active ETU (interactive gaming) will produce the highest engagement/arousal and screen exposure; passive ETU (neutral, low-arousal videos) will produce moderate-light engagement; and nondigital reading will be lowest on both. Accordingly, we hypothesize that active ETU has a greater impact on disrupting objective sleep and lower subjective sleep quality compared with passive ETU and nondigital technology nights.

Older adults are an important population for studying the underlying mechanisms related to sleep. Age-related changes in circadian and homeostatic regulation often lead to earlier sleep onset, reduced amplitude, and more fragmented sleep [34]. Consequently, factors like presleep arousal through the use of technology become especially significant. Additionally, changes in ocular and retinal function can affect spectral sensitivity, meaning the way older adults perceive light may differ from that of younger individuals [35].

Digital usage patterns among older adults also vary, typically involving more passive activities, such as viewing content, communicating, and playing simple games. These behaviors influence cognitive and emotional engagement [36]. Therefore, it is important, both demographically and conceptually, to investigate how different types of evening activities, such as those involving screen light and engaging content, affect sleep quality in older adults.

Unlike much of the existing research, which focuses primarily on children and adolescents [32,37], this study examines an underexplored demographic: older adults who are increasingly active users of digital technology and often have health issues linked to poor sleep.

### Methodological Consideration

This study will use a randomized crossover design. This within-subject approach limits confounding, minimizes the influence of inter-individual variability, and increases statistical power, which is especially important given the moderate sample size [3,4]. Objective sleep measures will be captured via a wearable EEG headband (MUSE), allowing for naturalistic, at-home sleep tracking that is both minimally intrusive and ecologically valid as it supports generalizability to real-world settings [29]. Additionally, participants will complete daily self-report ratings assessing their subjective sleep quality and mood. Single-item sleep quality measures have limitations because they lack the detail needed to capture the multi-dimensional nature of sleep problems, leading to issues with sensitivity, content validity, and reliability. However, the item is low-burden and has good construct validity [24]. The inclusion of a brief affective experience scale, adapted from validated instruments such as the Visual Analog Mood Scales [25], Positive and Negative Affect Schedule [26], and the User Engagement Scale [27], enables examination of psychological and experiential factors that may mediate the relationship between ETU type and sleep outcomes. Combining objective EEG data with subjective daily logs will provide a holistic, multidimensional understanding of sleep quality [18].

In this randomized crossover trial, participants will receive structured instructions and technical support to guide them in using the EEG headband and completing the assigned activities. These elements are designed to support compliance, usability, and data completeness. In this study design, there is a bias due to the carryover effect (residual effects of one condition influencing the next) and period/sequence effects (time- or order-related differences) [38]. These biases will be mitigated with a one-week washout period and by randomizing condition order, analyzing participant-level week means (which dampens night-to-night variability). This will also help to reduce weekend bias, as weekend sleep might differ from weekday sleep.

It is important to consider certain limitations of the study design and the expected results. Randomized crossover trials might increase the risk of participant dropout because, for a participant’s data to be included in the analysis, they must complete all intervention periods. To reduce attrition and support adherence, participants receive direct contact details (phone and email) for the lead author (SNG) and the university research clinic. They are also welcome to visit the clinic if they wish to discuss any issues. Recruitment and the initial information meeting are scheduled flexibly with 4 alternative time slots. Adherence is monitored via the daily log. If participants drop out before finishing all interventions, their data may be unusable, reducing the effective sample size and potentially introducing bias [39]. Although the crossover design helps control for inter-individual differences, subjective perceptions of each intervention may still influence self-reported sleep outcomes (eg, enjoyment or bias toward novelty). Nonadherence and usability challenges with the EEG device may affect the implementation of the intervention. Some participants may not follow the assigned activity consistently or may experience discomfort while using the MUSE EEG headband, which can impact their sleep. Technical issues or limited digital literacy could further reduce compliance or introduce usage bias [40]. Therefore, we will report the number of valid nights per condition and provide an attrition/participant flow summary in our results manuscript, allowing readers to assess compliance. These risks will be mitigated through initial training, daily logs, and reminders, but complete control is not guaranteed. Regarding the results, the findings will reflect the experiences of relatively healthy, digitally literate older adults and may not generalize to older populations with cognitive impairments or severe sleep disorders. Furthermore, the use of EEG headbands at home relies on participant compliance and technical reliability, which may affect data completeness.

### Future Research

This trial will be a first step toward clarifying the relationship between passive and active ETU and sleep in older adults. Findings should be interpreted with consideration for the short observation period and the emphasis on users experienced with digital technology. Future research could extend the duration of the intervention, include follow-ups, vary the “dose” and timing (eg, 15, 30, or 60 minutes, or the last hour before bed), and test device features such as blue light filters, night mode, and different types or paces of content. Implementation studies in clinics and community programs can assess the practicality, adherence, and effects of brief guidance on evening technology habits.

### Dissemination Plan

The findings from this trial will be disseminated through a peer-reviewed journal article, presentations at academic conferences, and promoted using social media.

### Conclusions

This protocol describes a crossover trial to evaluate the relationship between objective and subjective sleep and various types of evening activities, such as active technology use, passive technology use, and nondigital activities. If the hypothesized patterns are observed, the results could provide practical guidance on the use of presleep technology in late life. The findings may be useful in improving digital wellness programs for older adults, providing guidance on the timing, duration, and type of evening use.

## Supplementary material

10.2196/84512 Multimedia Appendix 1 Power analysis.

10.2196/84512 Multimedia Appendix 2 Baseline questionnaire–English.

10.2196/84512 Multimedia Appendix 3 Baseline questionnaire–Swedish.

## Abbreviations

EEG: electroencephalography
ETU: evening technology use
LMM: linear mixed-effects model
RCT: randomized controlled trial
REM: rapid eye movement
SE: sleep efficiency
SNAC-B: Swedish National Study on Aging and Care – Blekinge
SOL: sleep onset latency
TST: total sleep time
WASO: wake after sleep onset
