# Supplementary material for: The Effect of Evening Technology Use on Objective Sleep in Older Adults: Protocol for a Crossover Randomized Controlled Trial
Source: JMIR Res Protoc. 2026 Jan 30;15:e84512. doi: 10.2196/84512 (PMC12857899; doi:10.2196/84512)
Supplement: Multimedia Appendix 1 [file resprot-v15-e84512-s001.docx]

**Power analysis and calculating the intervention period**

Article;

Daily Electronic Media Use and Sleep in Late Life * https://doi.org/10.1093/geronb/gbae095

Leger et al. report a within-person coefficient for computer use predicting sleep duration of b = −0.44 and the SD of sleep duration is 1.15 (Table 1, page 4; Table 2, page 5). Treating the coefficient as the expected change across the daily range, a conservative standardized effect is:

dz ≈ 0.44 / 1.15 = 0.38.

We plan a crossover analysis using a linear mixed-effects model (LMM) with fixed effects for condition and period and a random participant intercept. The primary estimand is the within-participant contrast of combined ETU (Passive+Active averaged) versus Non-digital. For sample-size planning, we approximate this primary within-participant contrast using Cohen’s $d_{z}$ (standardized within-person difference), which provides a conservative sizing approach for the expected within-person effect.

Because there are two co-primary endpoints (SOL, WASO), we control the family-wise error at 0.05 using a Bonferroni adjustment; each two-sided primary test is evaluated at α = 0.025.

For a paired within-participant contrast using **dz**, the rough closed-form is:

A convenient sizing approximation uses **Cohen’s**dz (the standardized **within-person**effect):


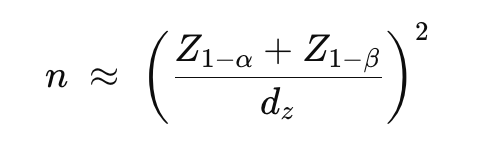


- **n** = required number of participants (pairs)
- Z1−αZ1−α​ = critical value for your two-sided alpha (e.g., with Bonferroni across two co-primary endpoints, α=0.025 two-sided ⇒ Z1−α/2 = Z0.9875 ≈ 2.24)
- Z1−βZ1−β​ = critical value for power (e.g., 0.842 for 80% power)
- dzdz​ = Cohen’s dzdz​ = (mean of paired differences) / (SD of paired differences)

n≈ 66

With n=50 completers at αadj=0.025 (two-sided) and dz=0.38, power ≈ 0.67 (under 0.80).

If α = 0.05 (two-sided) without Bonferroni adjustment, power = 80%

n≈ 55

Please note: Their outcome is self-reported sleep duration, not SOL/WASO. We use it only to set a conservative effect size for our paired contrast.
