# Supplementary material for: The Effect of Evening Technology Use on Objective Sleep in Older Adults: Protocol for a Crossover Randomized Controlled Trial
Source: JMIR Res Protoc. 2026 Jan 30;15:e84512. doi: 10.2196/84512 (PMC12857899; doi:10.2196/84512)
Supplement: Multimedia Appendix 2 [file resprot-v15-e84512-s002.docx]

| Gender  Baseline Questionnaire |  |
| --- | --- |
| Man | ☐ |
| Woman | ☐ |
| Who do you live with? |  |
| Alone | ☐ |
| Spouse/Partner | ☐ |
| What is the highest level of education you have completed? |  |
| Unfinished primary school | ☐ |
| Primary school | ☐ |
| Secondary school | ☐ |
| High school diploma or upper secondary school diploma | ☐ |
| Vocational training | ☐ |
| At least one year of education beyond upper secondary school (e.g. technical or specialised programme) | ☐ |
| University/college degree | ☐ |
| Doctoral degree (lic./dr) | ☐ |
| In summary, how would you describe your current financial situation? |  |
| Very good | ☐ |
| Quite good | ☐ |
| Neither good nor bad | ☐ |
| Pretty bad | ☐ |
| Very poor | ☐ |
| What is your current professional status? |  |
| Full-time work (30 hours or more per week) | ☐ |
| Part-time work (less than 30 hours per week, regularly scheduled) | ☐ |
| Retired (not working at all) | ☐ |
| Retired but occasionally working (irregular work, freelance or occasional short-term projects) | ☐ |
| Other (please specify) | ☐ |
| If you are currently employed, how many hours per week do you work? (Open-ended question) |  |
|  | |
| In general, would you say that your health is …? |  |
| Excellent | ☐ |
| Very Good | ☐ |
| Good | ☐ |
| Fair | ☐ |
| Bad | ☐ |

**Self-assessment of sleep health**

*Instruction: Check one option per row.*

| **Question** | **Rarely or never** | **Sometimes** | **Always or almost always** |
| --- | --- | --- | --- |
| Are you satisfied with your sleep? | ☐ | ☐ | ☐ |
| Do you stay awake all day without dozing off or taking a nap? | ☐ | ☐ | ☐ |
| Do you sleep, or try to sleep, between 2:00 and 4:00 a.m.? | ☐ | ☐ | ☐ |
| Are you awake less than 30 minutes per night? (this includes the time it takes to fall asleep and any awakenings) | ☐ | ☐ | ☐ |
| Do you sleep between 6 and 8 hours per 24 hours? | ☐ | ☐ | ☐ |

**Lifestyle behaviors / sleep habits (past month)**

*Instruction: Check one option per row.*

| **Question** | **Never** | **1–3 times / month** | **1–2 times / week** | **3–6 times / week** | **Every day** |
| --- | --- | --- | --- | --- | --- |
| Do you regularly do light exercise? (walks in streets/parks/forest, short bike rides, light aerobics, golf, etc.) | ☐ | ☐ | ☐ | ☐ | ☐ |
| Do you regularly do more intensive exercise? (jogging, brisk long walks, heavy gardening, long bike rides, intensive aerobics, long-distance skating, skiing, swimming, ball sports (not golf), etc.) | ☐ | ☐ | ☐ | ☐ | ☐ |
| Do you exercise within 2 hours before going to bed? | ☐ | ☐ | ☐ | ☐ | ☐ |
| Do you drink caffeinated beverages (coffee/tea/energy drink) later than 6 hours before bedtime? | ☐ | ☐ | ☐ | ☐ | ☐ |
| Do you wake up to use the bathroom two or more times during the night? | ☐ | ☐ | ☐ | ☐ | ☐ |
| Do you drink alcohol later than 6 hours before bedtime? | ☐ | ☐ | ☐ | ☐ | ☐ |
| Do you smoke or use snus later than 6 hours before bedtime? | ☐ | ☐ | ☐ | ☐ | ☐ |
| Do you eat a large meal within 3 hours before going to bed? | ☐ | ☐ | ☐ | ☐ | ☐ |
| Are you outdoors in daylight for more than 30 minutes per day? | ☐ | ☐ | ☐ | ☐ | ☐ |
| Do you take any medication to help you sleep? | ☐ | ☐ | ☐ | ☐ | ☐ |
| Do you wake up at the same time (within 1 hour) every day? | ☐ | ☐ | ☐ | ☐ | ☐ |
| Do you go to bed at the same time (within 1 hour) every day? | ☐ | ☐ | ☐ | ☐ | ☐ |
| Do you take a nap after 3:00 p.m.? | ☐ | ☐ | ☐ | ☐ | ☐ |
| Do you sleep more than 90 minutes during the day? | ☐ | ☐ | ☐ | ☐ | ☐ |
| Do you use screen-based technology (smartphone, computer, TV, other screens) within one hour before sleep? | ☐ | ☐ | ☐ | ☐ | ☐ |
| Do you have difficulty sleeping because of pain? | ☐ | ☐ | ☐ | ☐ | ☐ |
| Do you have difficulty sleeping because of itch? | ☐ | ☐ | ☐ | ☐ | ☐ |
| Do you have difficulty falling asleep/staying asleep because of mood? | ☐ | ☐ | ☐ | ☐ | ☐ |
| Do you have difficulty falling asleep/staying asleep because of stress? | ☐ | ☐ | ☐ | ☐ | ☐ |
| If you wake up at night, how often do you use your mobile phone (screen)? | ☐ | ☐ | ☐ | ☐ | ☐ |

# Internet use in the past three months

Instruction: For each device, check one option.

| **Device** | **Almost every day** | **At least once per week, but not every day** | **Less than once per week** | **Not at all** | **Have never used** |
| --- | --- | --- | --- | --- | --- |
| Desktop computer | ☐ | ☐ | ☐ | ☐ | ☐ |
| Laptop (notebook) | ☐ | ☐ | ☐ | ☐ | ☐ |
| Tablet / e-reader (e.g., iPad) | ☐ | ☐ | ☐ | ☐ | ☐ |
| Smartphone | ☐ | ☐ | ☐ | ☐ | ☐ |

# Reduced Morningness–Eveningness Questionnaire (rMEQ)

Instruction: For each question, tick one option.

### rMEQ-1. If you were entirely free to plan your day, about what time would you get up?

| **05:00–06:30** | **06:30–07:45** | **07:45–09:45** | **09:45–11:00** | **11:00–12:00** |
| --- | --- | --- | --- | --- |
| ☐ | ☐ | ☐ | ☐ | ☐ |

### rMEQ-2. How do you feel during the first half hour after waking up in the morning?

| **Very tired** | **Quite tired** | **Fairly alert** | **Very alert** |
| --- | --- | --- | --- |
| ☐ | ☐ | ☐ | ☐ |

### rMEQ-3. About what time in the evening do you feel tired and in need of sleep?

| **20:00–21:00** | **21:00–22:15** | **22:15–00:45** | **00:45–02:00** | **02:00–03:00** |
| --- | --- | --- | --- | --- |
| ☐ | ☐ | ☐ | ☐ | ☐ |

### rMEQ-4. At what time of day do you usually feel at your best?

| **05:00–08:00** | **08:00–10:00** | **10:00–17:00** | **17:00–22:00** | **22:00–05:00** |
| --- | --- | --- | --- | --- |
| ☐ | ☐ | ☐ | ☐ | ☐ |

### rMEQ-5. People sometimes talk about “morning types” and “evening types.” Which one do you consider yourself to be?

| **Definitely a morning type** | **More a morning than an evening type** | **More an evening than a morning type** | **Definitely an evening type** |
| --- | --- | --- | --- |
| ☐ | ☐ | ☐ | ☐ |

**DAILY LOG**


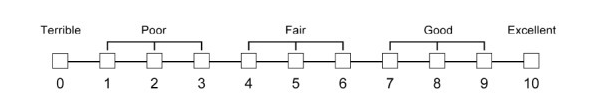
How would you rate your overall sleep quality? - Select only one box

# Intervention

Instruction: Tick ***one*** option and fill in if needed.

| **Question** | **Yes** | **No** | **Fell asleep during the intervention** |
| --- | --- | --- | --- |
| Were you able to complete the intervention? | ☐ | ☐ | ☐ |

**If yes, for how long?** __________________________________________

**If no, why?** _________________________________________________

# How did you experience the activity this evening?

Instruction: Rate each statement from ***0 (Not at all)*** to ***10 (Very much)***. Tick one box per row.

| **Statement** | **0** | **1** | **2** | **3** | **4** | **5** | **6** | **7** | **8** | **9** | **10** |
| --- | --- | --- | --- | --- | --- | --- | --- | --- | --- | --- | --- |
| I felt calm and relaxed. | ☐ | ☐ | ☐ | ☐ | ☐ | ☐ | ☐ | ☐ | ☐ | ☐ | ☐ |
| I felt stressed. | ☐ | ☐ | ☐ | ☐ | ☐ | ☐ | ☐ | ☐ | ☐ | ☐ | ☐ |
| I was mentally engaged in the activity. | ☐ | ☐ | ☐ | ☐ | ☐ | ☐ | ☐ | ☐ | ☐ | ☐ | ☐ |
| I enjoyed the activity. | ☐ | ☐ | ☐ | ☐ | ☐ | ☐ | ☐ | ☐ | ☐ | ☐ | ☐ |
| The activity felt meaningful to me. | ☐ | ☐ | ☐ | ☐ | ☐ | ☐ | ☐ | ☐ | ☐ | ☐ | ☐ |

# Comfort level

Question: “How comfortable do you find wearing the EEG headband during the night?”
Instruction: Tick one option.

| **1 – Very uncomfortable** | **2 – Somewhat uncomfortable** | **3 – Neither comfortable nor uncomfortable** | **4 – Somewhat comfortable** | **5 – Very comfortable** |
| --- | --- | --- | --- | --- |
| ☐ | ☐ | ☐ | ☐ | ☐ |

# Since last night, did anything unusual happen that could have affected your sleep?

**Answer (free text):**

You may include details such as: more caffeine than usual (esp. after ~16:00), alcohol, heavy/late meal, strong emotions/stress, late/intense exercise, long/late nap, pain/illness/allergy symptoms, new/missed/extra medications (incl. sleep meds), nicotine, travel or schedule changes, changes in noise/light/temperature, disturbances from partner/pets, screen use after lights out, etc.
