# Supplementary material for: The Effect of Evening Technology Use on Objective Sleep in Older Adults: Protocol for a Crossover Randomized Controlled Trial
Source: JMIR Res Protoc. 2026 Jan 30;15:e84512. doi: 10.2196/84512 (PMC12857899; doi:10.2196/84512)
Supplement: Multimedia Appendix 3 [file resprot-v15-e84512-s003.docx]

| Kön? | |
| --- | --- |
| Man | 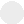 |
| Kvinna | 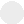 |

Baseline Questionnaire

| Vem eller vilka bor du  *tillsammans med? | |
| --- | --- |
| Ensamboende | 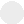 |
| Make/Maka/Sambo | 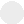 |
| Annan | 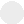 |
| Vilken är den högsta utbildningsnivå du har avslutat? |  |
| Oavslutad grundskola | 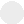 |
| Grundskola | 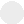 |
| Gymnasieskola | 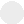 |
| Studentexamen eller gymnasieexamen | 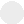 |
| Yrkesutbildning | 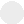 |
| Utbildning på minst ett år utöver gymnasiet (t.ex. tekniskt eller specialiserat program) | 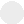 |
| Examen från universitet/högskola | 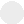 |
| Doktorsexamen (lic./dr) | 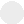 |

| Hur skulle du sammanfattningsvis beskriva din nuvarande ekonomiska situation? | |
| --- | --- |
| Mycket god | 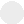 |
| Ganska god | 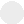 |
| Varrken god eller dålig | 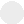 |
| Ganska dålig | 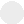 |
| Mycket dålig | 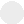 |

| Vad är din nuvarande yrkesmässiga status? | |
| --- | --- |
| Heltidsarbete (30 timmar eller mer per vecka) | 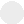 |
| Deltidsarbete (mindre än 30 timmar per vecka, regelbundet schemalagt) | 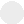 |
| Pensionär (arbetar inte alls) | 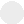 |
| Pensionerad men arbetar ibland (oregelbundet arbete, frilans eller enstaka kortsiktiga projekt) | 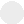 |
| Annat (vänligen specificera) | 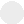 |

Om du arbetar för närvarande, hur många timmar per vecka arbetar du? (Öppet svar)

| I allmänhet skulle du vilja säga att din hälsa är …? | |
| --- | --- |
| Utmärkt | 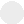 |
| Mycket God | 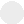 |
| God | 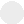 |
| Någorlunda | 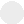 |
| Dålig | 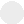 |

| **Här kommer några frågor om din sömnhälsa** | sällan eller aldrig | Ibland | alltid eller nästan alltid |
| --- | --- | --- | --- |
| Är du nöjd med din sömn? | 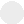 | 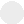 | 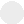 |
| Är du vaken hela dagen utan att slumra till eller ta en tupplur? | 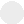 | 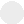 | 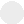 |
| Sover du, eller försöker du sova, mellan kl. 02 och 04 på natten? | 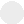 | 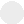 | 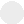 |
| Är du vaken mindre än 30 minuter per natt? (detta inkluderar den tid det tar att somna och eventuella uppvaknanden från sömnen) | 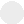 | 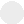 | 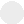 |
| Sover du mellan 6 och 8 timmar per dygn? | 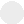 | 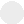 | 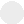 |

| **Livsstilsbeteenden/** **sömnvanor**  Följande frågor berör dina livsstilsbeteende/sömnvanor **den senaste månaden** | Aldrig | 1-3  ggr/  mån  - | 1-2  ggr/  vecka | 3-6  ggr/  vecka  - | Varje  dag |
| --- | --- | --- | --- | --- | --- |
| Motionerar du regelbundet med lättare motion?  *Promenader på vägar och i parker, skogspromenader, korta cykelturer, lätt gymnastik, golf eller liknande verksamhet* | 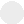 | 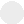 | 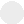 | 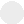 | 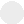 |
| Motionerar du regelbundet med mer intensiv motion?  *Jogging, raska långpromenader, tungt trädgårdsarbete, långa cykelturer, intensiv gymnastik, långfärdsskridskor, skidåkning, simning, bollspel (ej golf) eller annan liknande verksamhet* | 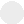 | 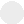 | 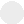 | 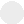 | 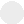 |
| Motionerar du under tidsperioden 2 timmar innan du går och lägger dig? | 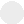 | 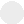 | 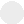 | 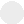 | 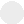 |
| Dricker du Koffeinhaltig dryck såsom kaffe/te/energidryck senare än 6 timmar innan du går och lägger dig? | 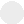 | 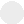 | 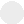 | 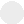 | 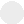 |
| Vaknar du för att gå på toaletten två eller fler gånger under natten? | 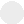 | 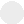 | 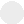 | 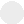 | 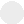 |
| Dricker du alkohol senare än 6 timmar innan du går och lägger dig? | 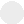 | 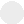 | 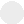 | 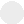 | 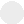 |
| Röker eller snusar du senare än 6 timmar innan du går och lägger dig? | 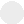 | 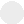 | 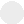 | 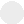 | 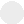 |
| Äter du en större måltid inom 3 timmar före du går och lägger dig för att sova? | 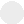 | 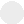 | 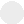 | 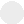 | 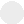 |
| Är du ute i dagsljus mer än 30 minuter per dag? | 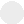 | 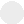 | 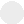 | 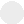 | 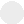 |
| Tar du något läkemedel för att hjälpa dig att sova? | 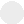 | 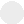 | 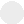 | 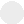 | 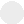 |
| Vaknar du vid samma tidpunkt (inom 1 timme) varje dag? | 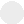 | 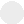 | 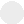 | 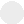 | 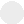 |
| Går du och lägger dig vid samma tidpunkt (inom 1 timme) varje dag? | 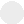 | 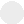 |  |  |  |
| Tar du en tupplur efter klockan 15.00? |  |  |  |  |  |
| Sover du mer än 90 minuter dagtid? |  |  |  |  |  |
| Använder du teknik med skärm (smartphone, dator, TV eller andra skärmar) inom en timme före du sover? |  |  |  |  |  |
| Har du svårigheter att sova på grund av smärta? |  |  |  |  |  |
| Har du svårigheter att sova på grund av klåda? |  |  |  |  |  |
| Har du svårigheter att somna/förbli sovande på grund av sinnesstämning? |  |  |  |  |  |
| Har du svårigheter att somna/förbli sovande på grund av stress? |  |  |  |  |  |
| Hur ofta använder du mobiltelefon med skärm om du vaknar på natten? |  |  |  |  |  |
| Vaknar du för att gå på toaletten två eller fler gånger under natten? |  |  |  |  |  |

| **A1. Hur ofta har du i genomsnitt använt INTERNET i dator, tablet och smartphone under de senaste tre månaderna?** | | | | | |
| --- | --- | --- | --- | --- | --- |
|  | I stort sett varje dag | Minst en gång per vecka, men inte varje dag | Mindre än en gång per vecka | Inte alls | Har aldrig använt |
| Stationär dator |  |  |  |  |  |
| Bärbar dator, laptop |  |  |  |  |  |
| Tablet, läsplatta (t.ex) iPad |  |  |  |  |  |
| Smartphone |  |  |  |  |  |

**The reduced Morningness–Eveningness Questionnaire**

rMEQ-1. Ungefär vilken tid skulle du gå upp om du var helt fri att planera din dag?
• [5] kl. 05.00–06.30
• [4] kl. 06.30–07.45
• [3] kl. 07.45–09.45
• [2] kl. 09.45–11.00
• [1] kl. 11.00–12.00

rMEQ-2. Hur känner du dig under den första halvtimmen efter att du har vaknat på morgonen?
• [1] Mycket trött
• [2] Ganska trött
• [3] Ganska pigg
• [4] Mycket pigg

rMEQ-3. Vid ungefär vilken tid på kvällen känner du dig trött och i behov av sömn?
• [5] kl. 20.00–21.00
• [4] kl. 21.00–22.15
• [3] kl. 22.15–00.45
• [2] kl. 00.45–02.00
• [1] kl. 02.00–03.00

rMEQ-4. Vid ungefär vilken tid på dygnet känner du dig vanligtvis som piggast?
• [5] kl. 05.00–08.00
• [4] kl. 08.00–10.00
• [3] kl. 10.00–17.00
• [2] kl. 17.00–22.00
• [1] kl. 22.00–05.00

rMEQ-5. Man talar ibland om “morgontyper” och “kvällstyper.” Vilken av dessa typer anser du att du är?
• [6] Definitivt en morgontyp
• [4] Snarare mer en morgontyp än en kvällstyp
• [2] Snarare mer en kvällstyp än en morgontyp
• [0] Definitivt en kvällstyp

**DAILY LOG**

SLEEP: Single-Item Sleep Quality Scale

(doi: [10.5664/jcsm.7478](https://doi.org/10.5664/jcsm.7478))

Hur kommer du att bedöma din sömnkvalitet totalt sett - Välj endast en ruta

Intervention
Fråga: Kunde du fullfölja interventionen?

Ja

Nej

Somnade under interventionen

Om ja, hur länge?
Om nej, Varför?

**Hur upplevde du aktiviteten i kväll?**

Vänligen betygsätt varje påstående från **0 (Inte alls)** till **10 (I högsta grad)**.

1. Jag kände mig lugn och avslappnad.
   0 ────────────────────────── 5 ────────────────────────── 10
2. Jag kände mig stressad.
   0 ────────────────────────── 5 ────────────────────────── 10
3. Jag var mentalt engagerad i aktiviteten.
   0 ────────────────────────── 5 ────────────────────────── 10
4. Jag tyckte om aktiviteten.
   0 ────────────────────────── 5 ────────────────────────── 10
5. Aktiviteten kändes meningsfull för mig.
   0 ────────────────────────── 5 ────────────────────────── 10

Komfortnivå
”Hur bekvämt tycker du att det är att bära EEG-pannbandet under natten?”
1 – Mycket obekvämt
2 – Något obekvämt
3 – Varken bekvämt eller obekvämt
4 – Något bekvämt
5 – Mycket bekvämt

**Sedan i går kväll, hände något ovanligt som kan ha påverkat din sömn?**
*(Om ja, beskriv kortfattat.)*
**Exempel:** mer koffein än vanligt (kaffe/te/energidrycker, särskilt efter ca kl. 16); alkohol; tung eller sen måltid; starka känslor/stress; sen eller intensiv träning; längre/sen tupplur; smärta, sjukdom eller allergisymtom; nya, missade eller extra läkemedel (inkl. sömnmedel); nikotin; resa eller förändrat schema (senare/tidigare läggdags); förändringar i ljud/ljus/temperatur; störningar från partner/husdjur; skärmanvändning efter släckning; etc.
